# Reference Data of Phase Angle Using Bioelectrical Impedance Analysis in Overweight and Obese Chinese

**DOI:** 10.3389/fendo.2022.924199

**Published:** 2022-07-12

**Authors:** Luo Fu, Zhengyun Ren, Xiaoxiao Liu, Nianwei Wu, Kang Zhao, Guangping Luo, Huawu Yang, Yuanchuan Zhang, Tong Yan, Yanjun Liu, Tongtong Zhang

**Affiliations:** ^1^ Center of Gastrointestinal and Minimally Invasive Surgery, Department of General Surgery, The Third People’s Hospital of Chengdu, Affiliated Hospital of Southwest Jiaotong University & The Second Affiliated Hospital of Chengdu, Chongqing Medical University, Chengdu, China; ^2^ College of Medicine, Southwest Jiaotong University, Chengdu, China; ^3^ Medical Research Center, The Third People’s Hospital of Chengdu, Affiliated Hospital of Southwest Jiaotong University & The Second Affiliated Hospital of Chengdu, Chongqing Medical University, Chengdu, China

**Keywords:** phase angle, reference data, bioelectrical impedance analysis, SMF-BIA, overweight and obese Chinese

## Abstract

**Introduction:**

Phase angle (PhA) is a ratio of reactance and resistance {arctangent (reactance (Xc)/resistance (R)) × (180°/π)}, which can be obtained by bioelectrical impedance analysis (BIA). PhA indicates cellular health and integrity, and it is also considered as a prognostic tool in medical disorders and an indicator of nutritional status (especially of muscle quality) in patients with obesity. However, PhA has limited usefulness in clinical practice because of a lackness of reference values for Chinese overweight and obese populations. The main aim of this study was to show PhA reference data in different age and BMI groups by sex. In addition, we also study the association of age, sex, and BMI on PhA.

**Methods:**

A total of 1729 overweight and obese participants were included in this study. PhA and body composition were measured using segmental multifrequency BIA. Differences in mean values for variables were tested by one-way analysis of variance. Multiple regression analysis was used to assess the associations of PhA with age, sex and BMI.

**Results:**

Multiple regression analysis showed that age, sex and BMI were significant (*P* < 0.05) independent influence factors of PhA in Chinese overweight and obese adults when age and BMI were continues variables. The mean PhA value for all participants was 5.5°. Mean BMI, age, weight, height and 50kHz-PhA were significantly higher (*P* < 0.001) in male participants than female ones. In age groups and BMI groups, mean 50kHz-PhA was significantly higher (*P* < 0.005) in male participants than female ones. When age groups and BMI groups were categorical variables, multiple regression analysis showed that different age groups (46–55 years and ≥ 56 years) had a significantly lower (*P* < 0.005) PhA as compared with the baseline group (18-25 years) and different BMI groups (≥ 28 kg/m^2^) had a significantly higher (*P* < 0.05) PhA as compared with the baseline group (24–27.9 kg/m^2^).

**Conclusion:**

PhA differed according to age, sex and BMI. Reference data in this study can be taken into consideration when deriving the reference values for overweight and obese Chinese populations.

## Introduction

Phase angle (PhA), a ratio of reactance and resistance {arctangent (reactance (Xc)/resistance (R)) × (180°/π)}, which can be obtained by bioelectrical impedance analysis (BIA), is the difference between voltage and current. PhA is a non-invasive indicator that can be used to predict nutritional status, total body water, and the intracellular and extracellular water distribution (1–4). The higher the PhA value (PhA refers to PhA at 50 kHz unless otherwise specified), the healthier the cells with intact cell membranes in the human body; the lower the PhA value, the more damage to the function of cells and integrity of the cell membrane (5). Disease, inflammation, dysfunction, malnutrition, and unhealthy lifestyle habits can lead to disturbances in the electrical properties of tissues, which can affect the PhA (6–8). In contrast to other body composition measures that use BIA, PhA is not affected by different regression equations or the assumption of constant tissue hydration (5).

BIA is a convenient, non-invasive, and inexpensive method for assessing body composition, and it is an attractive diagnostic tool for clinical and research settings (9). The basis of the method is impedance (Z, the body’s opposition to alternating current), which is determined *via* two components: resistance (R, the resistance of the human body to electrical current, inversely proportional to water and electrolytes) and reactance (Xc, capacitive resistance, depending on the function and integrity of the body cell mass) (10, 11). Traditional BIA systems are influenced by the composition of different body regions. It has been reported that BIA is strongly dependent on muscle mass in the distal extremities, and it cannot accurately detect fluid changes within the trunk (12). Compared with traditional BIA, segmental multifrequency BIA (SMF-BIA) overcomes inconsistencies between resistance (R) and body mass of the trunk (10). Other than that, SMF-BIA has a convenience from measurements of this modality usually being made with stand-on devices. SMF-BIA divides the body area according to limbs and trunk and can obtain the resistance and reactance values of several different frequency currents. However, there are little data on the use of SMF-BIA to measure PhA in overweight and obese people.

The World Health Organization (WHO) defines overweight and obesity as abnormal or excessive fat accumulation that presents a risk to health. According to the “Report on Nutrition and Chronic Disease Status of Chinese Residents (2020),” more than half of adults in China are overweight/obese, and the rates of overweight/obesity among children under 6 years of age and those aged 6–17 years are 19.0% and 10.4%, respectively (13). Overweight and obesity increase the risk of multiple chronic diseases in adults and can lead to serious heart and cerebrovascular diseases as well as endocrine and metabolic disorders. Overweight and obesity may also cause respiratory, digestive, and motor system disorders and are related to the occurrence of various malignant tumors (14, 15).

Obesity not only increases adipose tissue but also alters the structure, function, and metabolic characteristics of skeletal muscle (16). The combination of obesity and reduced muscle function is associated with greater disability (17) risk and lower survival (18). Intermuscular and intramuscular fat infiltration, which are directly linked to excess body fat, increase with age and adiposity (19–21). The PhA has a direct relationship to muscle strength (22, 23); the PhA value is higher in athletes (24) and lower in individuals with sarcopenia (25). Thus, the European Working Group on Sarcopenia in Older People 2019 consensus on sarcopenia proposed PhA as a possible index of muscle quality (26).

There is currently no PhA reference value for overweight and obese Chinese people that can be used for clinical and scientific research, and there are few studies using SMF-BIA to measure PhA in overweight and obese people. Considering that PhA values vary by age, sex, ethnicity, hydration, nutrition, and body composition (27, 28), it is necessary to study the PhA reference in Chinese overweight and obese populations. The main aim of this study was to show PhA reference data in difference age and BMI groups by sex. In addition, we also study the association of age, sex, and BMI on PhA.

## Material and Methods

### Participants

The study was conducted on 1813 overweight and obese participants who attended a physical examination at the Third People’s Hospital of Chengdu, China between November 2020 and September 2021. Of 1813 participants, we excluded 84 participants aged < 18 years old. As a retrospective study the data were anonymous; the requirement for informed consent was therefore waived. The study protocol and informed consent procedure were approved by the institutional ethics committee under registration number 2018-S-75. All procedures performed in studies involving human participants were in accordance with the ethical standards of institutional and/or national research committee and with the 1964 Declaration of Helsinki and its later amendments or with comparable ethical standards.

### Anthropometric Data

Data for each participant were recorded in a uniform format. Anthropometric measurements were made according to the standards established by the WHO. For weight calculation, participants wore light clothing and measurement was conducted using a SMF-BIA device (Inbody770, Biospace, Korea) to the nearest 0.1 kg. Standing height was measured using a stadiometer to the nearest 0.1 cm. BMI was calculated as weight in kilograms divided by the square of height in meters (kg/m^2^).

### Bioelectric Impedance Measurement

Participants were measured by an advanced SMF-BIA device (Inbody770, Biospace, Korea) following standard procedures (29), according to the manufacturer’s instructions. Participants were asked to stand barefoot on the analyzer, hold the handle lightly with both hands, kept their arms straight to ensure the arms did not touch the sides of the body, and kept the thighs from touching each other. The participants’ thumbs were placed on the top oval electrode of the handle, and the remaining four fingers were placed on the bottom electrode. The heel was aligned with the rear foot electrode. The PhA is directly measured using the inbuilt formula: PhA = arctangent (reactance (Xc)/resistance (R)) × (180°/π) (30, 31). The basic principle of body composition analysis used by Inbody770 is to assume that the human body has four main components (water, protein, mineral, and fat) and divide body water into two parts: intracellular water and extracellular water. SMF-BIA takes into account that the body consists of five heterogeneous cylinders (trunk and four limbs). The predictive methods used by Inbody770 are proprietary information and largely unknown. They are presumed to be based on regression models.

The measurement results of SMF-BIA are affected by many factors, such as hydration status, diet, body position, air humidity, skin temperature, and physical activity (27, 32). To control these factors, before test, it should be ensured that individuals have an empty stomach and bladder and have not recently engaged in strenuous exercise. In addition, the individual should stand still for approximately 5 minutes before measurement to maintain a stable water distribution in the body. In this study, all participants were guided by professionals during the test to ensure that they were properly prepared and in the the correct test posture so as to obtain accurate test results.

### Statistical Analysis

Continuous variables are expressed as mean ± standard deviation and categorical variables are expressed as number and percentage. Descriptive characteristics of the study population were stratified by sex, and analysis of variance was used to study the differences in population characteristics by sex.

We exhibited the PhA reference data in term of mean ± standard deviation. Multiple regression analysis was used to assess the associations of PhA with age, sex and BMI. We also conducted independent influence factors analysis for Chinese 50kHz-phase angle when age groups and BMI groups were categorical variables. We adjusted age (18-25 years, 26–35 years, 36–45 years, 46–55 years, and ≥ 56 years) and BMI (24.0–27.9 kg/m^2^, 28.0–31.9 kg/m^2^, 32.0–35.9 kg/m^2^, 36.0–39.9 kg/m^2^, and ≥ 40 kg/m^2^) as categorical variables instead of continues variables. Parametric statistics were used in all above analysis due to the distribution of PhA was fairly normal in our participants (*P* for normality test based on Skewness and kurtosis tests = 0.214).

We also presented PhA data for each body segment (right arm, left arm, trunk, right leg and left leg) in difference age and BMI groups by sex. The percent body fat (PBF) and skeletal muscle mass (SMM) for participants were also be presented in the same way. All analyses were conducted using Stata 16.0 (Stata Corp LLC, College Station, TX, USA). We used 50 kHz whole-body PhA as the PhA value in the study. *P*<0.05 was defined as statistically significant.

## Results

### Descriptive Characteristics

A total of 1729 overweight and obese participants were included in the study. Among them, 330 were overweight with BMI between 24.0 and 27.9 kg/m^2^, and 1399 were obese with a BMI ≥ 28 kg/m^2^. The mean PhA value was 5.5 ± 0.6°. The characteristics (age, weight, height, BMI and 50kHz-PhA) of the study population were stratified by sex, shown in **Table 1**. Phase angle was significantly larger in the men (n=542, 31%) than in the women (5.9 ± 0.7° and 5.3 ± 0.5°, respectively; *P* < 0.001). Mean BMI, age, weight and height were significantly higher (*P* < 0.001) in male participants than female ones.

**Table 1:** Age, weight, height, BMI, and 50kHz-phase angle of the participants by sex.

Characteristics	Men	Women	Total	P^*^
	(N = 542)	(N = 1187)	(N = 1729)	
Age (years)	36.0 ± 12.5	33.9 ± 9.6	34.6 ± 10.7	<0.001
Weight (kg)	104.7 ± 24.2	85.5 ± 16.6	91.5 ± 21.2	<0.001
Height (cm)	172.8 ± 7.0	160.5 ± 6.0	164.4 ± 8.5	<0.001
BMI (kg/m^2^)	34.9 ± 7.2	33.1 ± 6.0	33.7 ± 6.4	<0.001
50kHz-PhA (°)	5.9 ± 0.7	5.3 ± 0.5	5.5 ± 0.6	<0.001

BMI, body mass index; SD, standard deviation; PhA, Phase angle.

All values were Mean ± SD.

^*^P by ANOVA.

### Multiple Regression Analysis

We conducted independent influence factors analysis for Chinese 50kHz-PhA when age and BMI were continues variables, as shown in **Table 2**. Multiple regression analysis showed that age, sex and BMI were significant (*P* < 0.05) independent influence factors of PhA in Chinese overweight and obese adults. After controlling for the effect of gender and BMI on PhA, PhA showed a negative correlation (*P* < 0.001) with age. We also conducted independent influence factors analysis for Chinese 50kHz-PhA when age groups and BMI groups were categorical variables, as shown in **Table 3**. Multiple regression analysis showed that different age groups (46–55 years and ≥ 56 years) had a significantly lower (*P* < 0.005) PhA as compared with the baseline group (18-25 years) and different BMI groups (≥28 kg/m^2^) had a significantly higher (*P* < 0.05) PhA as compared with the baseline group (24–27.9 kg/m^2^). The PhA of the participants at 26-35 years and 36-45 years was not statistically different from the baseline group (18-25 years), so PhA was not a complete negative correlation with age.

**Table 2:** Independent influence factors analysis for Chinese 50kHz-phase angle when age and BMI as continues variables.

Variables	β	SE	*P*	95% CI	
				lower	upper
Sex (ref: Women)					
Men	0.629	0.029	0.000	0.572	0.687
Age (years)	-0.014	0.001	0.000	-0.016	-0.011
BMI (kg/m^2^)	0.006	0.002	0.010	0.001	0.010

BMI, body mass index; CI, confidence interval; SE, standard error.

**Table 3:** Independent influence factors analysis for Chinese 50kHz-phase angle when age groups and BMI groups as categorical variables.

Variables	β	SE	*P*	95% CI	
				lower	upper
Sex (ref: Women)					
Men	0.645	0.028	0.000	0.589	0.701
Age groups (ref: 18-25 years)					
26-35 years	0.067	0.037	0.069	-0.005	0.139
36-45 years	0.050	0.042	0.229	-0.032	0.131
46-55 years	-0.196	0.057	0.001	-0.307	-0.084
≥56 years	-0.718	0.069	0.000	-0.853	-0.582
BMI groups (ref: 24-27.9 kg/m^2^)					
28-31.9 kg/m^2^	0.133	0.040	0.001	0.055	0.211
32-35.9 kg/m^2^	0.227	0.040	0.000	0.148	0.306
36-39.9 kg/m^2^	0.288	0.046	0.000	0.199	0.378
≥40 kg/m^2^	0.128	0.046	0.006	0.037	0.220

BMI, body mass index; CI, confidence interval; SE, standard error.

### PhA in Age and BMI Groups

Because BMI and age were significantly associated with phase angle in the previous analysis, it is necessary to group by BMI and age to further study the effect of BMI and age on PhA. The 50kHz-PhA for Chinese in difference age and BMI groups stratified by sex was shown in **Table 4**. In difference age groups and BMI groups, mean 50kHz-PhA was significantly higher (*P* < 0.005) in male participants than female ones. We also presented PhA data for each body segment (right arm, left arm, trunk, right leg and left leg) in difference age and BMI groups, stratified by sex, as shown in **Tables S1** **–** **S5**. We found that males had significantly higher (*P* < 0.001) mean PhA than females in all BMI groups no matter which body segment. The PhA was significantly greater (*P* < 0.05) in males than in females in all age groups no matter which body segment except for PhA of right leg in subjects ≥ 56 years.

**Table 4:** 50kHz-phase angle for Chinese in difference age and BMI groups by sex.

Variables	Men		Women		P^*^
	N	Mean ± SD	N	Mean ± SD	
**Age groups**					
18-25 years	94	6.1 ± 0.6	199	5.3 ± 0.5	0.000
26-35 years	225	6.0 ± 0.6	587	5.4 ± 0.5	0.000
36-45 years	131	6.0 ± 0.6	276	5.4 ± 0.5	0.000
46-55 years	48	5.8 ± 0.7	85	5.1 ± 0.5	0.000
≥56 years	44	5.1 ± 0.7	40	4.6 ± 0.5	0.002
**BMI groups**					
24-27.9 kg/m^2^	99	5.7 ± 0.8	231	5.1 ± 0.5	0.000
28-31.9 kg/m^2^	107	5.8 ± 0.6	335	5.3 ± 0.5	0.000
32-35.9 kg/m^2^	118	6.0 ± 0.6	324	5.4 ± 0.5	0.000
36-39.9 kg/m^2^	108	6.2 ± 0.5	156	5.5 ± 0.5	0.000
≥40 kg/m^2^	110	5.9 ± 0.6	141	5.4 ± 0.5	0.000

BMI, body mass index; SD, standard deviation.

^*^P by ANOVA.

### PBF and SMM

The mean PBF and SMM were presented in difference age and BMI groups, stratified by sex, as shown in **Tables S6** **,** **S7**. The females had significantly higher (*P* < 0.005) mean PBF and significantly lower (*P* < 0.005) mean SMM than males in all age and BMI groups.

## Discussion

The aim of this study was to examine the influence of sex, age and body mass index (BMI) on 50kHz-PhA, and to establish 50kHz-PhA reference data for overweight and obese Chinese adults. In addition, we presented PhA data for each body segment (right arm, left arm, trunk, right leg and left leg) in difference age and BMI groups by sex. The percent body fat (PBF) and skeletal muscle mass (SMM) for participants were also be presented in the same way.

The estimated mean PhA in this study was 5.5 ± 0.6°, and the mean PhA for overweight men and women is 5.7 ± 0.8° and 5.1 ± 0.5°, respectively. A study in China found similar results. Guangya Chen et al. (33), in the study of PhA and nonalcoholic fatty liver disease, found that participants (BMI ≥ 24) had a mean PhA of 5.50 ± 0.65°. The mean PhA in our study is significantly smaller than the mean PhA reported in Europe and the United States, indicating that ethnicity has a large effect on PhA (27, 28, 30, 34, 35). We found significant sex differences in PhA, with PhA values significantly higher (*P* < 0.001) in male than in female participants. Males have been found to have higher PhA (36–40), which may be related to greater fat-free mass (lower resistance) (41, 42). However, some studies have reported no difference in the mean PhA value between male and female individuals (30, 43), which may be caused by a small sample size.

Our findings confirmed that the PhA of baseline group (18-25 years) were significantly higher (*P* < 0.005) as compared with the participants aged ≥ 46 years, while the PhA of the participants at 26-35 years and 36-45 years was not statistically different from the baseline group (18-25 years). The only study on the matter found that PhA was decreased after the fourth decade of life (40). Because age was not grouped or the effect from other variables on PhA was not controlled, some studies have found a negative relationship between age and PhA (34, 35, 41, 44), although within each age group the effect of age on PhA is not the same. It is worth noting that PhA may be altered in severely obese individuals with changes in body cell mass and tissue hydration (40). Thus, further research of PhA is needed specifically on people who are overweight and obese.

Many studies decompose BMI into two variables of height and weight for research (45, 46). Considering that the purpose of this study was to investigate overweight and obese people according to BMI, we only studied the effect of BMI on PhA. One study took waist circumference into account, and severely obese patients (BMI ≥ 35 kg/m^2^) with higher BMI and waist circumference had lower PhA (47). Of note, although there is no definite conclusion regarding the impact of excess body fat on PhA (48), the PhA was inversely related to fat mass (49) and directly related to fat-free mass (50). We discovered that the PhA showed a positive correlation with BMI and different BMI groups (≥28 kg/m^2^) had a significantly higher (*P* < 0.05) PhA as compared with the baseline group (24–27.9 kg/m^2^). One study reported that PhA was lower in people with class III obesity (BMI: 40-64 kg/m^2^) than those with class I/II obesity (BMI: 30-35 kg/m^2^) (51). In addition, a paper reported that PhA increased with increased BMI (≤ 35 kg/m^2^), decreased with BMI > 35 kg/m^2^, and PhA was negatively correlated with BMI regardless of sex, with BMI > 40kg/m^2^ (40). Conversely, another study showed that PhA did not differ across BMI subgroups (30–39.9 kg/m^2^, 40–49.9 kg/m^2^, and ≥ 50 kg/m^2^) (38).

The limitations of this study include the following: The results of the present study only apply to overweight and obese people and the sample may be not representative because the study only included data from Chengdu, China. Owing to data limitations, we could not obtain additional variables that may affect the PhA, so there may be some confounding factors that we could not control.

In summary, in this study, we assessed the influence of sex, age and BMI on 50kHz-PhA, and provided important new reference data for PhA for overweight and obese Chinese adults by the SMF-BIA method. These data can be used for clinical practice and research settings. Based on current study of PhA, we can consider that next steps include increasing the sample size and enhance the representativeness of the sample in future research to establish reference values for PhA in overweight and obese Chinese adults. The relationship between PhA and body compositions and the use of PhA in nutritional assessment and disease prognosis is worthy of attention. It is worth noting that there are no unified standards for measurement methods (selection of instrument, current intensity, frequency, calculation formula, and operating procedures), and PhA measured using the new BIA technique lacks comparison with the older technique. Solving these problems requires ongoing research efforts.

## Data Availability Statement

The raw data supporting the conclusions of this article will be made available by the authors, without undue reservation.

## Ethics Statement

The studies involving human participants were reviewed and approved by Ethics Committee of Chengdu Third People’s Hospital. Written informed consent for participation was not required for this study in accordance with the national legislation and the institutional requirements.

## Author Contributions

Conception and design of study: LF, ZR, XL, KZ. Acquisition of data: LF, ZR,YZ, YL. Analysis and/or interpretation of data: NW, XL, KZ. Drafting the manuscript: LF, ZR, GL, HY, TZ. Revising the manuscript critically for important intellectual content: TZ, XL, NW, KZ, TY, YL. Approval of the version of the manuscript to be published: LF, ZR, XL, NW, KZ, GL, HY, YZ, TY, YL, TZ. All authors contributed to the article and approved the submitted version.

## Funding

This work was supported by Science and Technology Department of Chengdu (2021-YF05-00103-SN), Science and Technology Project of the Health Planning Committee of Sichuan (19PJ012) and National Natural Science Foundation of China (82170887).

## Conflict of Interest

The authors declare that the research was conducted in the absence of any commercial or financial relationships that could be construed as a potential conflict of interest.

## Publisher’s Note

All claims expressed in this article are solely those of the authors and do not necessarily represent those of their affiliated organizations, or those of the publisher, the editors and the reviewers. Any product that may be evaluated in this article, or claim that may be made by its manufacturer, is not guaranteed or endorsed by the publisher.
