# Supplementary material for: Reference Data of Phase Angle Using Bioelectrical Impedance Analysis in Overweight and Obese Chinese
Source: Front Endocrinol (Lausanne). 2022 Jul 12;13:924199. doi: 10.3389/fendo.2022.924199 (PMC9319044; doi:10.3389/fendo.2022.924199)
Supplement: Supplementary file 1 [file Table_1.pdf]

Table S1. 50kHz-phase angle of right arm for Chinese in difference age and BMI groups by sex.

Variables	Men		Women		P^*
	N	Mean ± SD	N	Mean ± SD	
**Age groups**					
18-25 years	94	5.5 ± 0.6	199	4.7 ± 0.5	0.000
26-35 years	225	5.6 ± 0.6	587	4.9 ± 0.5	0.000
36-45 years	131	5.7 ± 0.6	276	5.0 ± 0.5	0.000
46-55 years	48	5.5 ± 0.7	85	4.8 ± 0.6	0.000
≥56 years	44	5.0 ± 0.6	40	4.4 ± 0.5	0.000
**BMI groups**					
24-27.9 kg/m^2	99	5.4 ± 0.7	231	4.6 ± 0.5	0.000
28-31.9 kg/m^2	107	5.5 ± 0.6	335	4.9 ± 0.5	0.000
32-35.9 kg/m^2	118	5.6 ± 0.6	324	5.0 ± 0.5	0.000
36-39.9 kg/m^2	108	5.7 ± 0.5	156	5.0 ± 0.5	0.000
≥40 kg/m^2	110	5.5 ± 0.6	141	5.0 ± 0.5	0.000

Abbreviations: BMI, body mass index; SD, standard deviation.

$^*P$ by ANOVA.
