# Supplementary material for: Reference Data of Phase Angle Using Bioelectrical Impedance Analysis in Overweight and Obese Chinese
Source: Front Endocrinol (Lausanne). 2022 Jul 12;13:924199. doi: 10.3389/fendo.2022.924199 (PMC9319044; doi:10.3389/fendo.2022.924199)
Supplement: Supplementary file 3 [file Table_3.pdf]

Table S3. 50kHz-phase angle of trunk for Chinese in difference age and BMI groups by sex.

**Variables**	**Men**		**Women**		P^*
	**N**	**Mean ± SD**	**N**	**Mean ± SD**	
**Age groups**					
18-25 years	94	7.8 ± 0.9	199	6.8 ± 0.8	0.000
26-35 years	225	7.4 ± 1.1	587	6.7 ± 0.8	0.000
36-45 years	131	7.3 ± 1.1	276	6.6 ± 0.8	0.000
46-55 years	48	7.1 ± 1.0	85	6.3 ± 0.8	0.000
≥56 years	44	6.4 ± 0.9	40	5.9 ± 0.8	0.021
**BMI groups**					
24-27.9 kg/m^2	99	7.2 ± 1.1	231	6.5 ± 0.8	0.000
28-31.9 kg/m^2	107	7.3 ± 1.0	335	6.6 ± 0.8	0.000
32-35.9 kg/m^2	118	7.5 ± 1.0	324	6.7 ± 0.8	0.000
36-39.9 kg/m^2	108	7.5 ± 0.9	156	6.6 ± 0.8	0.000
≥40 kg/m^2	110	7.1 ± 1.2	141	6.5 ± 0.8	0.000

Abbreviations: BMI, body mass index; SD, standard deviation.

$^*P$ by ANOVA.
