# Supplementary material for: Reference Data of Phase Angle Using Bioelectrical Impedance Analysis in Overweight and Obese Chinese
Source: Front Endocrinol (Lausanne). 2022 Jul 12;13:924199. doi: 10.3389/fendo.2022.924199 (PMC9319044; doi:10.3389/fendo.2022.924199)
Supplement: Supplementary file 4 [file Table_4.pdf]

Table S4. 50kHz-phase angle of right leg for Chinese in difference age and BMI groups by sex.

Variables	Men		Women		P^*
	N	Mean ± SD	N	Mean ± SD	
**Age groups**					
18-25 years	94	6.7 ± 0.7	199	6.0 ± 0.6	0.000
26-35 years	225	6.5 ± 0.7	587	6.0 ± 0.6	0.000
36-45 years	131	6.3 ± 0.8	276	5.8 ± 0.6	0.000
46-55 years	48	5.9 ± 0.9	85	5.4 ± 0.6	0.000
≥56 years	44	5.0 ± 0.8	40	4.7 ± 0.7	0.083
**BMI groups**					
24-27.9 kg/m^2	99	6.0 ± 1.0	231	5.6 ± 0.7	0.000
28-31.9 kg/m^2	107	6.2 ± 0.8	335	5.8 ± 0.7	0.000
32-35.9 kg/m^2	118	6.4 ± 0.8	324	6.0 ± 0.6	0.000
36-39.9 kg/m^2	108	6.6 ± 0.6	156	6.0 ± 0.6	0.000
≥40 kg/m^2	110	6.3 ± 0.8	141	6.0 ± 0.6	0.000

Abbreviations: BMI, body mass index; SD, standard deviation.
$^*P$ by ANOVA.
