# Supplementary material for: Reference Data of Phase Angle Using Bioelectrical Impedance Analysis in Overweight and Obese Chinese
Source: Front Endocrinol (Lausanne). 2022 Jul 12;13:924199. doi: 10.3389/fendo.2022.924199 (PMC9319044; doi:10.3389/fendo.2022.924199)
Supplement: Supplementary file 6 [file Table_6.pdf]

Table S6. Percent body fat for Chinese in difference age and BMI groups by sex.

**Variables**	**Men**		**Women**		P^*
	**N**	**Mean ± SD**	**N**	**Mean ± SD**	
**Age groups**					
18-25 years	94	38.6 ± 8.0	199	45.1 ± 5.8	0.000
26-35 years	225	37.2 ± 7.3	587	43.8 ± 5.6	0.000
36-45 years	131	35.6 ± 7.9	276	42.7 ± 5.3	0.000
46-55 years	48	33.5 ± 6.0	85	42.0 ± 5.6	0.000
≥56 years	44	30.9 ± 4.3	40	41.9 ± 6.1	0.002
**BMI groups**					
24-27.9 kg/m^2	99	26.6 ± 4.8	231	36.9 ± 3.9	0.000
28-31.9 kg/m^2	107	32.3 ± 4.0	335	41.3 ± 3.5	0.000
32-35.9 kg/m^2	118	36.2 ± 3.6	324	45.1 ± 3.2	0.000
36-39.9 kg/m^2	108	39.3 ± 3.3	156	47.9 ± 3.0	0.000
≥40 kg/m^2	110	45.8 ± 4.6	141	51.5 ± 3.0	0.000

Abbreviations: BMI, body mass index; SD, standard deviation.

$^*P$ by ANOVA.
