# Supplementary material for: Reference Data of Phase Angle Using Bioelectrical Impedance Analysis in Overweight and Obese Chinese
Source: Front Endocrinol (Lausanne). 2022 Jul 12;13:924199. doi: 10.3389/fendo.2022.924199 (PMC9319044; doi:10.3389/fendo.2022.924199)
Supplement: Supplementary file 7 [file Table_7.pdf]

Table S7. Skeletal muscle mass for Chinese in difference age and BMI groups by sex.

**Variables**	**Men**		**Women**		***P***[*]
	**N**	**Mean ± SD**	**N**	**Mean ± SD**	
**Age groups**					
18-25 years	94	38.5 ± 5.4	199	27.0 ± 3.8	0.000
26-35 years	225	38.7 ± 5.3	587	26.4 ± 3.8	0.000
36-45 years	131	36.7 ± 5.4	276	26.1 ± 3.3	0.000
46-55 years	48	33.3 ± 4.3	85	25.2 ± 3.0	0.000
≥56 years	44	28.1 ± 3.6	40	21.8 ± 3.8	0.002
**BMI groups**					
24-27.9 kg/m^2	99	31.1 ± 4.2	231	22.9 ± 2.8	0.000
28-31.9 kg/m^2	107	33.5 ± 4.4	335	25.0 ± 2.6	0.000
32-35.9 kg/m^2	118	37.2 ± 4.2	324	26.7 ± 2.8	0.000
36-39.9 kg/m^2	108	39.8 ± 4.1	156	28.3 ± 3.0	0.000
≥40 kg/m^2	110	42.1 ± 5.3	141	30.7 ± 4.0	0.000

Abbreviations: BMI, body mass index; SD, standard deviation.

[*]*P* by ANOVA.
